# Comparable Infection Level and Tropism of Measles Virus and Canine Distemper Virus in Organotypic Brain Slice Cultures Obtained from Natural Host Species

**DOI:** 10.3390/v13081582

**Published:** 2021-08-10

**Authors:** Brigitta M. Laksono, Diana N. Tran, Ivanela Kondova, Harry G. H. van Engelen, Samira Michels, Sham Nambulli, Rory D. de Vries, W. Paul Duprex, Georges M. G. M. Verjans, Rik L. de Swart

**Affiliations:** 1 Department of Viroscience, Erasmus MC, 3015 GD Rotterdam, The Netherlands; b.laksono@erasmusmc.nl (B.M.L.); d.tran@erasmusmc.nl (D.N.T.); s.michels@erasmusmc.nl (S.M.); r.d.devries@erasmusmc.nl (R.D.d.V.); g.verjans@erasmusmc.nl (G.M.G.M.V.); 2 Division of Pathology, Animal Science Department, Biomedical Primate Research Centre, 2280 GH Rijswijk, The Netherlands; kondova@bprc.nl; 3 Department of Clinical Sciences of Companion Animals, Veterinary Medicine, Universiteit Utrecht, 3584 CM Utrecht, The Netherlands; h.g.h.vanengelen@uu.nl; 4 Centre for Vaccine Research, University of Pittsburgh School of Medicine, Pittsburgh, PA 15261, USA; nambulli@pitt.edu (S.N.); pduprex@pitt.edu (W.P.D.)

**Keywords:** measles virus, canine distemper virus, morbillivirus, organotypic brain slice culture, central nervous system, pathogenesis, tropism

## Abstract

Measles virus (MV) and canine distemper virus (CDV) are closely related members of the family *Paramyxoviridae*, genus *Morbillivirus*. MV infection of humans and non-human primates (NHPs) results in a self-limiting disease, which rarely involves central nervous system (CNS) complications. In contrast, infection of carnivores with CDV usually results in severe disease, in which CNS complications are common and the case-fatality rate is high. To compare the neurovirulence and neurotropism of MV and CDV, we established a short-term organotypic brain slice culture system of the olfactory bulb, hippocampus, or cortex obtained from NHPs, dogs, and ferrets. Slices were inoculated ex vivo with wild-type-based recombinant CDV or MV expressing a fluorescent reporter protein. The infection level of both morbilliviruses was determined at different times post-infection. We observed equivalent infection levels and identified microglia as main target cells in CDV-inoculated carnivore and MV-inoculated NHP brain tissue slices. Neurons were also susceptible to MV infection in NHP brain slice cultures. Our findings suggest that MV and CDV have comparable neurotropism and intrinsic capacity to infect CNS-resident cells of their natural host species.

## 1. Introduction

Measles virus (MV) and canine distemper virus (CDV) are enveloped viruses with an unsegmented negative sense single-stranded RNA genome that belong to the family *Paramyxoviridae,* genus *Morbillivirus* [1]. MV causes disease in humans and non-human primates (NHPs), while CDV infects a broad range of species, including dogs, raccoons, mustelids, lions, and even macaques [2,3,4,5,6,7]. Acute measles is characterised by fever, cough, and maculopapular rash, which are usually resolved within a week. Case-fatality rates are low, and morbidity and mortality most often result from measles-associated immune suppression [8]. CDV infection in their natural hosts, in contrast, often leads to severe disease, associated with high case-fatality rates [9]. Both viruses are also known to cause neurological complications, but CNS infection is strikingly much more frequent in canine distemper than in measles. 

Morbillivirus-associated neurological complications vary in their disease progressions and presentations. Measles inclusion body encephalitis (MIBE) can develop early after acute measles in patients with pre-existing immune deficiencies [10,11]. Long-term MV persistence and host inability to clear the virus may lead to subacute sclerosing panencephalitis (SSPE). SSPE is rare with an estimated incidence rate of 1 in 10,000 cases, which increases to 1 in 600 in children who had measles under the age of 12 months [12,13]. Onset of SSPE symptoms usually occurs five to ten years after measles [14]. SSPE symptoms include behavioural problems and personality changes, seizure, cognitive dysfunction, and ultimately coma [15]. To date, there is no effective treatment for SSPE, and the disease is always fatal. The case-fatality rate of CDV infection depends on the species infected, from 50% in dogs to close to 100% in ferrets, regardless of the age of the animals [9]. CDV often causes acute or subacute meningoencephalitis in its natural host species [16,17], which may be considered a counterpart of MIBE in humans. In addition, CDV can cause old dog encephalitis, which is considered the closest counterpart to SSPE—the neurological symptoms can appear years after infection [18]. 

Various animal models have been developed to study the pathogenesis of morbillivirus brain infection, but most rely on unnatural inoculation routes with laboratory-adapted virus strains in animal species that are not naturally susceptible to morbillivirus infection [10,19,20]. In the last decade, ex vivo organotypic brain slice culture has become a promising tool to study CNS infection. Unlike immortalised cell lines, brain slices maintain cell–cell interactions and tissue structure that closely resemble the in vivo situation. Organotypic brain slice cultures were initially developed to study human neurological and psychiatric diseases [21,22]. The cultures have also been used in morbillivirus infection studies, but exclusively using rodent tissues [23,24,25,26]. Rodents are not naturally susceptible to wild-type MV infection and hence these models poorly represent MV-associated CNS infection in humans [10,19,20]. The organotypic brain slice culture offers a new way to study the tropism of morbilliviruses in the CNS, especially in the early stage of the infection [27]. Brain endothelial cells, neurons, and glial cells, for example, have been suggested to be the target cells of morbillivirus infection in the brain [27,28,29,30].

To study the neurovirulence and neurotropism in the early stage of MV and CDV infection, we have used organotypic brain slice cultures of naturally susceptible NHPs, dogs, and ferrets. We selected olfactory bulb, hippocampal, and cortical tissues to be included in this study, based on where infected cells have been detected in CDV- and MV-infected brains [31,32,33]. The use of recombinant CDV and MV, based on wild-type strains and expressing fluorescent reporter proteins, allowed us to sensitively monitor the progression of infection and characterise the phenotypes of the infected cells. We included the highly neurotropic CDV strain Snyder Hill (SH), which was passaged in dog brains [17]; the wild-type CDV strain Rhode Island, which was isolated from a raccoon [34]; two MV strains IC323 and Khartoum-Sudan (KS) which were based on wild-type MV strains [35,36]. We demonstrate comparable infection levels and tropism of these morbilliviruses in their natural host species’ brain slice cultures.

## 2. Materials and Methods

### 2.1. Ethical Statement

This study exclusively used surplus tissues from animal experiments unrelated to this study, and no animals were euthanised specifically for this study. Post-mortem dog (*Canis lupus familiaris*; *n* = 3; 1 year old) brain tissues were donated by the Faculty of Veterinary Medicine in Utrecht University, Utrecht, the Netherlands. Post-mortem rhesus macaque (*Macaca mulatta*; *n* = 6; 10–18 years old) brain tissues were dissected and obtained from the Biomedical Primate Research Centre, Rijswijk, the Netherlands. Post-mortem ferret (*Mustela putorius furo*; *n* = 3; approximately 1 year old) brain tissues were donated by the Department of Viroscience, Erasmus MC, Rotterdam, the Netherlands. 

### 2.2. Tissue Collection and Transport

Brain was dissected in ice-cold Hank’s balanced salt solution (HBSS) supplemented with sucrose [37]. A piece of the frontal cortex, hippocampi, and olfactory bulbs were stored in Hibernate-A medium (Gibco, Paisley, UK; A12475–01) during transport at room temperature [21,22]. Tissues were processed within 2 to 4 h after euthanasia.

### 2.3. Viruses and Cells

All viruses were recombinant (r) viruses expressing enhanced green fluorescent protein (EGFP) or Venus as reporter protein from an additional transcription unit within different positions (position 1, 3, or 6) of the genome. Two different strains of CDV were included in this study: CDV strain Rhode Island expressing reporter protein Venus at position 6 of the viral genome (rCDV^RI^Venus(6); TCID_50_: 1.7 × 10^6^/mL) [34] and CDV strain Snyder Hill expressing EGFP at position 6 of the viral genome (rCDV^SH^EGFP(6); TCID_50_: 2.6 × 10^6^/mL) [17]. The rCDV^RI^Venus(6) is a recombinant virus based on a wild-type virus isolated from a raccoon in Rhode Island, while rCDV^SH^EGFP(6) is a laboratory-adapted, highly neurovirulent strain based on a natural CDV isolated from dogs passaged in vivo in dog brains. Two strains of MV were included in this study: MV strain Khartoum-Sudan expressing the reporter EGFP at position 3 of the viral genome (rMV^KS^EGFP(3); TCID_50_: 3.7 × 10^6^/mL), and MV strain IC323 expressing EGFP at position 1 of the viral genome (rMV^IC323^EGFP(1); TCID_50_: 2.7 × 10^6^/mL). CDV stocks were grown on Vero-dogSLAM (VDS) cells, a kind gift from Dr Y. Yanagi (Kyushu University, Fukuoka, Japan) [38]. The rMV^KS^EGFP(3) and rMV^IC323^EGFP(1) were grown on Epstein–Barr virus-transformed B lymphoblastoid cell line (BLCL) [36] and Vero-humanSLAM (VHS) cells [35], respectively. BLCL were grown in RPMI-1640 [39] and the VHS cells were grown in Dulbecco’s modified Eagle medium. All culture media were supplemented with 10% foetal bovine serum, 100 IU/mL of penicillin, 100 µg/mL of streptomycin, and 2 mM glutamine. VDS and VHS cells are Vero cells expressing the dog or human SLAMF1 (signalling lymphocyte activation marker family member 1; also known as CD150) receptor. 

### 2.4. Brain Slice Culture 

The frontal cortex (approximate dimension of 2 × 2 × 2 cm^3^), olfactory bulb, and hippocampus tissues were cut into 300 µm thick slices with a McIlwain tissue slicer (Oss Life Science Park, Oss, the Netherlands). To allow tissues to recover from the mechanical damage, the slices were incubated in non-supplemented Hibernate-A medium with 5% CO_2_ for 2 h at 37 °C. To assess how long ex vivo brain slices can remain viable upon slicing and culturing, uninfected dog, ferret, or rhesus macaque brain slices were kept free-floating in culture for 8 days in a 48-well plate in Neurobasal-A medium, which contains D-glucose, (Gibco, New York, NY, USA; 10888-022) supplemented with 20 µL/mL B27 (Gibco, New York, NY, USA, 17504-044), 10 µL/mL glutaMAX, 1 µL/mL gentamycin, and 2 µL/mL HEPES buffer. The brain tissues were placed in a mixture of propidium iodide (PI) (0.22 mg/L) and phosphate buffered saline (PBS) for 2 min and immediately washed 3 times with PBS prior to detection of fluorescent signal using an inverted confocal laser scanning microscope (Zeiss LSM700, Carl Zeiss, Jena, Germany). The staining was performed at day 0, 1, 2, 3, and 8 post-slicing and based on observation on the number of cell deaths, the subsequent brain slices were cultured for no longer than 3 days. The remaining tissues were inoculated with cell-free viruses (200 µL/well) in a 24-well plate at 37 °C with 5% CO_2_ for 2 h. Carnivore tissues slices were inoculated with rCDV^RI^Venus(6), rCDV^SH^EGFP(6), or rMV^KS^EGFP(3). NHP tissue slices were inoculated with rMV^IC323^EGFP(1), rMV^KS^EGFP(3), or rCDV^RI^Venus(6). After inoculation, the slices were incubated in a 48-well plate (1 slice per well) with the aforementioned Neurobasal-A medium (the slices floated freely in the medium) at 37 °C with 5% CO_2_ for 3 days. All inoculated tissues were monitored and scored daily under an inverted laser scanning microscope for fluorescent cells. Tissues with Venus^+^ or EGFP^+^ cells were fixed in freshly prepared, methanol-free 4% PFA for further analysis. Grading of the degree of morbillivirus infection in ex vivo brain slice cultures was performed semi-quantitatively. Low level of infection (+) was defined when a single infected cell was observed in one brain slice; moderate level (++) when up to 10 infected cells were observed, and high level (+++) when >10 infected cells were observed.

### 2.5. Indirect Immunofluorescence Labelling on Whole Mount Brain Tissue Slices

Phenotypes of infected cells in the brain tissue slices were determined by immunofluorescent labelling for neuron-specific marker NeuN, astrocyte-specific marker glial fibrillary acidic protein (GFAP), microglia-specific marker ionised calcium binding adaptor molecule 1 (Iba1), or oligodendrocyte-specific marker 2′,3′-cyclic-nucleotide 3′-phosphodiestrase (CNPase). We also stained for β-3 tubulin (tubIII) to identify neurons, but were unable reproducibly stain neurons in all species. Cortex, olfactory bulb, and hippocampus slices were washed with PBS and, except for NeuN and CNPase stainings, antigen retrieval was conducted by heating the slices at 95 °C in citrate buffer for 20 min. Blocking was performed in PBS with 10% donkey serum, 0.5% TritonX-100, and 0.2% gelatine solution. Tissues were first incubated with mouse monoclonal anti-human NeuN (Sigma Aldrich, Amsterdam, the Netherlands, MAB377, 1:250), mouse monoclonal anti-human GFAP (Sigma Aldrich, Amsterdam, the Netherlands, G3893, 1:400), or rabbit anti-human Iba1 (Wako, Neuss, Germany, LKG5732, 1:250). The slices were incubated with primary antibodies overnight at 4 °C in PBS with 10% of donkey serum, 0.1% of Tween-20 and 0.2% of gelatine solution. After a wash with PBS with 0.1% of Tween-20, the slices were incubated overnight at 4 °C with goat anti-rabbit Alexa Fluor 555 (Invitrogen, Landsmeer, the Netherlands, A32732, 1:250) or polyclonal goat anti-mouse RPE (Dako, Santa Clara, USA, R0480, 1:250) in combination with goat anti-GFP FITC (Abcam, Amsterdam, the Netherlands, ab6662, 1:250). Unbound antibodies were washed away with PBS with 0.1% of Tween-20. The tissues were incubated with Hoechst 33342 (1:600,000) for 10 min prior to detection of fluorescent signals using an inverted confocal laser scanning microscope. The anti-human NeuN antibody successfully cross-reacted with NHP tissues, but not carnivore ones.

## 3. Results

### 3.1. Establishment of an Organotypic Brain Slice Culture System from Adult Ferrets, Dogs, and NHPs

We established an organotypic brain slice culture from adult ferrets, dogs, and NHPs, based on protocols described in previous studies [21,22]. Olfactory bulb, hippocampus, and frontal cortex were collected during necropsy immediately after euthanasia, placed in medium, and cut into 300 µm thick slices. To optimise culture conditions, we cultured the free-floating slices up to 8 days. One slice was harvested each day and treated with PI to monitor the tissue viability. Under our current protocol, the brain slice cultures remained viable for at least 3 days after processing. Based on this observation, we performed the subsequent morbillivirus infection experiment up to 3 days post-inoculation (dpi).

### 3.2. Organotypic Brain Slices of Ferrets, Dogs, and NHPs Are Susceptible and Permissive to Ex Vivo Morbillivirus Infection

To assess whether carnivore brain slices are susceptible to CDV infection, we inoculated olfactory bulb, cortex, and hippocampal slices (*n* = 4 per tissue per time point) obtained from ferrets (*n* = 3) or dogs (*n* = 3) with cell-free recombinant CDV strain RI or SH expressing the fluorescent reporter protein Venus (rCDV^RI^Venus(6)) or enhanced green fluorescent protein (rCDV^SH^EGFP(6)). rMV^KS^EGFP(3) was included as a control virus in these experiments. CDV-infected cells were observed in all brain tissues from 2 dpi onwards, a time span which was in accordance with previous observations in CDV-infected primary tissues [40]. Inoculation with rMV^KS^EGFP(3) resulted in no or limited infection in ferret and dog brain slices. Since viral infection in the brain slices was focal and varied between tissues and species, we developed a scoring system to assess the infection levels. In ferret and, to a lesser degree, dog brain slice cultures, CDV-infected cells were present in highest numbers in the olfactory bulb slices (Table 1). Interestingly, we found more rCDV^SH^EGFP(6)-infected cells in ferret brain tissues, especially the olfactory bulb, than in dog tissues, despite its high neurovirulence in both ferrets and dogs in vivo [17]. Based on these observations, we concluded that the carnivore brain slices were susceptible and permissive to ex vivo CDV infection.

To assess whether NHP brain slices are susceptible to MV infection, we inoculated olfactory bulb, cortex, and hippocampal slices (*n* = 4 per tissue per time point) obtained from rhesus macaques (*n* = 6) with rMV^KS^EGFP(3) or rMV^IC323^EGFP(1) and recorded the progression of infection up to 3 dpi. rCDV^RI^Venus(6) was included as a control virus in these experiments. Similar to the observations in CDV-inoculated carnivore brain slices, we observed MV-infected cells in primate brain slices from 2 dpi onwards, a time span which was in accordance with previous observations of MV infection on primary cells or tissues [40,41] (Table 2). However, in NHP brain slice cultures, the highest numbers of infected cells were detected in the cortex. Inoculation with rCDV^RI^Venus(6) resulted in no or limited infection in NHP brain slices.

### 3.3. Different Morphology of Morbillivirus-Infected Cells in Organotypic Brain Slice Cultures of Ferrets, Dogs, and NHPs

**Figure 1:**
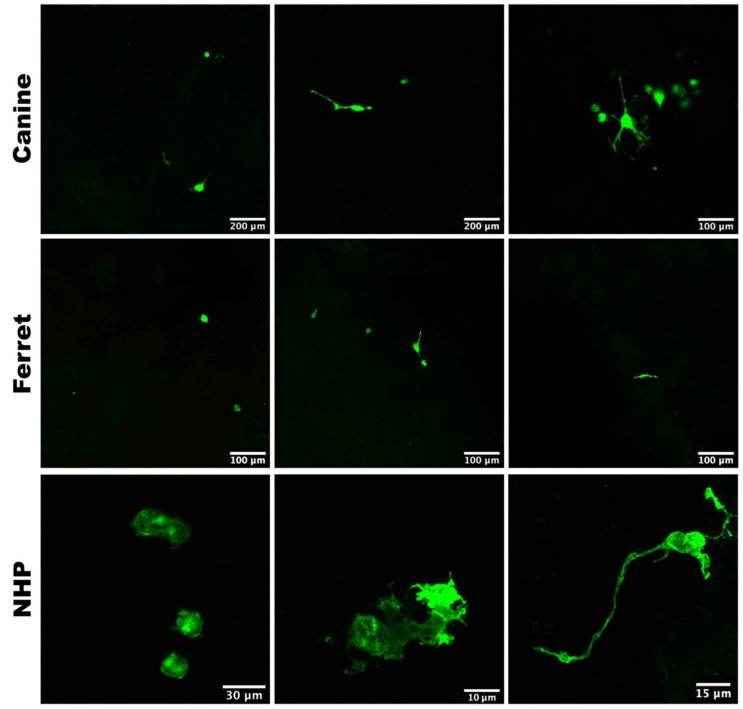
Different morphology of morbillivirus-infected cells. Whereas most virus-infected cells (green) were round single cells (left column), cells with different morphology were also observed in CDV-infected dog and ferret, and MV-infected NHP brain slices (middle and right columns).

We observed different morphologies of CDV- and MV-infected cells in their respective natural host brain slice cultures. Most CDV- and MV-infected cells appeared to be in majority round single cells, while some infected cells showed dendrite-like protrusions (Figure 1). Interestingly, there was no evidence of disseminated infection in the surrounding neuropil of the brain slices, even in tissues inoculated with the highly neurovirulent rCDV^SH^EGFP(6). Rather, some of the infected cells that were detectable at 2 dpi could no longer be found at 3 dpi. The disappearance of the infected cells was most often observed on slices with only few infected cells per slice.

### 3.4. Microglia, Neurons, and Oligodendrocytes Are the Main Susceptible Cell Types to Morbillivirus Infection

**Figure 2:**
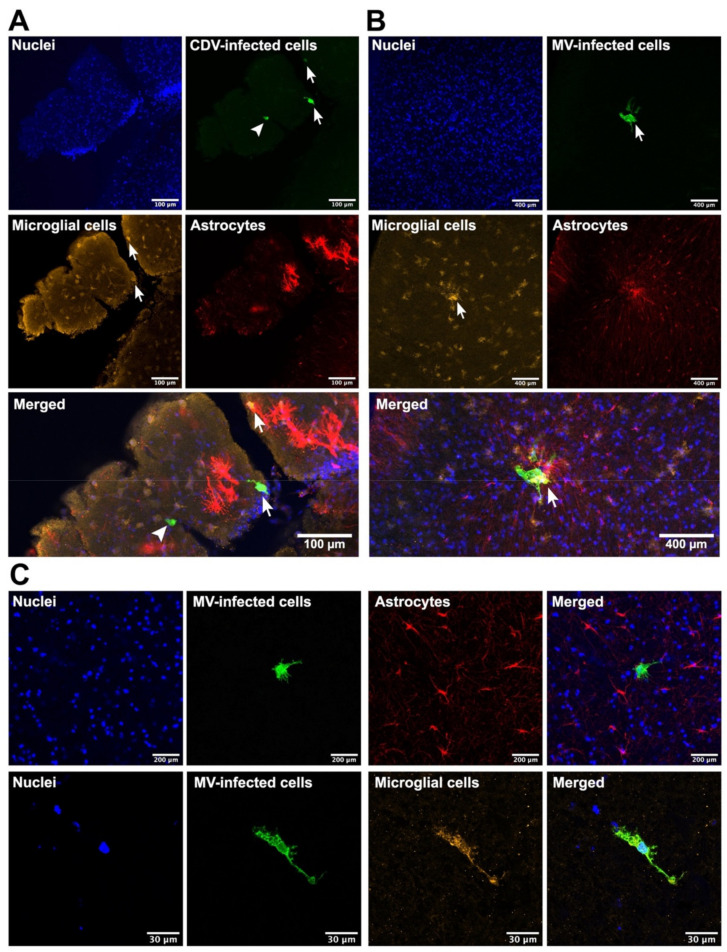
Microglia, but not astrocytes, in brain slice cultures were susceptible to morbillivirus infection. (**A**) Representative CDV-infected ferret hippocampal slices at 2 days post-infection (dpi). Some CDV-infected cells (green, arrows) were microglia (Iba1^+^ cells; yellow), but not astrocytes (GFAP^+^ cells, red). An Iba1^−^ GFAP^−^ CDV-infected cell (green, arrowhead) was also present. (**B**,**C**) Representative ex vivo NHP cortex slices. MV-infected cells (green, arrows) were mainly microglia (yellow), but not astrocytes (red).

**Figure 3:**
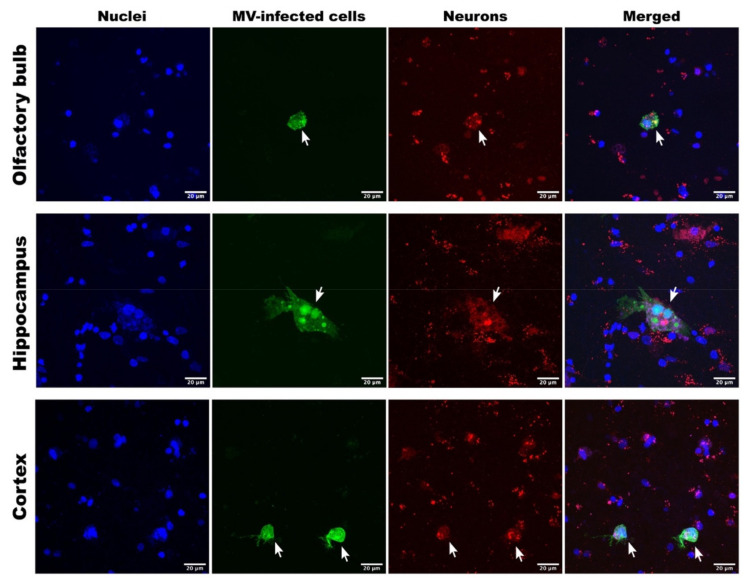
Neurons in NHP brain slice cultures were susceptible to MV infection. Representative MV-infected (green) NHP neurons (NeuN^+^; red) in the olfactory bulb, hippocampal, and cortex slices collected at 3 days post-inoculation.

**Figure 4:**
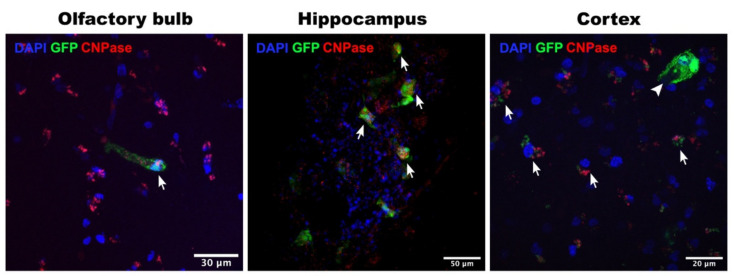
Oligodendrocytes in NHP brain slice cultures were susceptible to MV infection. Representative MV-infected (GFP^+^; green) NHP oligodendrocytes (CNPase^+^; red) in the olfactory bulb, hippocampal, and cortex slices. Olfactory bulb and cortex slices were collected at 3 days post-inoculation (dpi) and hippocampal slice was collected at 4 dpi. In some slices, infected cells that were CNPase^−^ were also present, as represented in the cortex slice (arrowhead).

To determine the phenotypes of the CDV- or MV-infected cells, we performed dual-immunofluorescent (IF) staining on the CDV- or MV-inoculated carnivore or primate brain slices, respectively. We identified astrocytes, microglia, and neurons in carnivore and NHP brain slices based on their cellular markers: glial fibrillic acidic protein (GFAP), ionised calcium binding adaptor molecule 1 (Iba1), and NeuN proteins, respectively. In carnivore brain slices, we only succeeded in identifying GFAP^+^ astrocytes and Iba1^+^ microglia, due to lack of cross-reactivity of the antibody to carnivore NeuN proteins. Venus or EGFP expression co-localised with Iba1 staining in carnivore and NHP brain slices (Figure 2), indicating that microglia were infected by CDV and MV. Astrocytes were observed in close proximity to virus-infected cells, but were never infected. In NHP brain slices, we observed co-localisation of Venus or EGFP with NeuN staining, signifying MV-infected neurons (Figure 3). Oligodendrocytes were also infected by MV in these NHP brain slices in all observed tissues (Figure 4). Altogether, our findings show that MV and CDV display equivalent intrinsic neurotropism and neurovirulence.

## 4. Discussion

In this study, we inoculated organotypic brain slice cultures with wild-type-based recombinant CDV or MV expressing fluorescent reporter proteins to study morbillivirus brain infection. Surprisingly, we observed comparable levels of MV and CDV infection in brain slices of primates and carnivores, respectively. Moreover, we found that both morbilliviruses predominantly infected microglia. Although we could not assess if CDV-infected carnivore neurons due to the lack of cross-reactive antibodies, we observed MV-infected neurons in NHP brain slices. We also detected MV-infected oligodendrocytes in these NHP brain slices. These cells may also be infected by CDV in dog and ferret brain slices, since we also observed the presence of CDV-infected Iba1^−^ GFAP^−^ cells in ferret brain slices. The infection of microglia, neurons, and oligodendrocytes was in accordance with previous observations in in vivo MV and CDV CNS infection [27,30]. Interestingly, astrocytes were not susceptible to morbillivirus infection, suggesting that at the early stage of morbillivirus CNS infection, these cells are not the primary target cells. Altogether, these findings suggest that the intrinsic neurovirulence and neurotropism of MV and CDV may be comparable.

We observed differences in the susceptibility of tissues collected from different parts of the brain to ex vivo morbillivirus infection. In carnivores, more CDV-infected cells were found in the olfactory bulb relative to other tissues. In NHPs, the cortex slice cultures tended to harbour higher numbers of MV-infected cells. These tissue-related differences may partly be influenced by different distribution and density of susceptible cells, especially microglia. In humans, the ratio of glia-to-neuron is higher in cerebral cortex than in the cerebellum [42]. This high density of microglia in human cortex could be similar in NHPs. Whether there is a high density of microglia in the olfactory bulbs of carnivores remains to be determined. It is not known if microglia, like macrophages, express CD150, although a recent study has reported that the expression of CD150 on microglia is inducible [43]. CD150, expressed by immune cells and nectin-4, expressed on the basolateral side of epithelial cells, are the entry receptors of wild-type MV and CDV [44,45,46]. Myeloid cells are considered primary target cells during the early stage of morbillivirus infections [47]. Microglia arise from the progenitor cells in the embryonic yolk sac. They are in close vicinity to neurons and share similar features with cells of myeloid origin, which are susceptible to morbillivirus infection. Viremia after morbillivirus infection is mostly cell-associated and the infection is predominantly disseminated by circulating or migrating infected T and B cells [48,49]. Ferrets infected with highly neurovirulent CDV showed that the virus invaded through two distinct pathways: via the cribriform plate (by direct infection of the olfactory nerves) or via the haematogenous route (the choroid plexus and cerebral blood vessels) [17,50]. The haematogenous infection spreads into the CNS through CDV-infected lymphocytes and myeloid cells, consistent with the high level of viremia during canine distemper. The same route of dissemination into the CNS may be applicable to but less efficient for MV, since the number of MV-infected cells in the circulation is lower than that of CDV [51]. Based on these observations, in combination with the fact that these immune cells can pass the blood–brain barrier [52,53], we hypothesise that morbillivirus-infected lymphocytes bring the virus into the CNS and can transmit it to microglia. In the majority of the cases, the infection is likely controlled by the host immune system. In some cases the virus can obtain hyperfusogenic mutations allowing it to spread from microglia to neuron and subsequently from neuron to neuron. Spread into neurons can be facilitated independent of CD150 or nectin-4 by *cis-*interaction of CADM1 and CADM2 on infected neurons, allowing virus transmission to interacting neurons [54]. Alternatively, spread into neurons can be dependent of nectin-4 through transfers of cytoplasmic cargo from infected epithelial cells to nectin-1-expressing neurons [55].

We demonstrate that CDV and MV infection were most prominent in carnivore and primate brain slices, respectively. However, we also noticed low levels of infected cells in the heterologous brain slices. It has been well documented that MV can use dog CD150 [56], which could potentially explain the observed MV infection of dog and, to a lesser extent, ferret brain slices. CDV cannot use human CD150 as a cellular receptor in vitro, but has caused outbreaks in NHPs [6,57] and was shown to be able to use NHP CD150 in vitro [58]. In vivo inoculation of NHPs with CDV also resulted in productive but self-limiting infection, thus showing that CDV in NHPs behaves more similarly to MV in NHPs than CDV in carnivores [59]. Nevertheless, we observed only low level CDV infection in NHP brain slices.

SSPE can take years to develop after recovery from measles, but it is not known exactly when MV reaches the brain. In a study of 717 measles cases in children, 95% did not show any clinical evidence of encephalitis. However, 344 patients showed abnormally slow electroencephalograms during or immediately after the acute phase of the disease [60]. CNS infection in measles thus may be a more common feature than expected and likely is contained before developing or presenting clinical evidence of encephalitis. MV RNA has also been detected in the brains of autopsied individuals with no SSPE-like CNS complication or history [61,62]. Altogether, these findings indicate that MV can potentially invade the CNS more often than previously thought and persist long after the acute phase of disease. However, our current organotypic brain slice culture model, although permissive, could not sustain MV or CDV infection, thus not allowing us to observe the persistence of these viruses in our model. Persistence of MV may lead to accumulation of mutations in the matrix (M) or the fusion (F) gene. Hypermutation in the M gene results in the lack of virus particle formation while mutations in the F gene lead to hyperfusogenicity. In our study, neurons of NHPs were susceptible to ex vivo MV infection, but the infection did not spread and, at times, could even not be detected the following day, suggesting lytic infection or rapid local innate immune clearance. We also did not observe cell-to-cell spread in carnivore brain slice culture inoculated with rCDV^SH^EGFP(6), despite the virus possessing the necessary mutations. Morbillivirus ribonucleoprotein (RNP) complex is transmitted at the synapses, allowing membrane fusion to happen only in such area [63]. We speculate that synaptic transmission between neurons in our ex vivo brain model may be disrupted after slicing, hence preventing transneuronal spread of viral RNP complex. The regulation of synaptic functions in the CNS is also supported by endocrine hormones, which was absent in our organotypic brain slice culture. This may explain the unexpected observation that ex vivo infection with the highly neurotropic CDV strain SH was less efficient than infection with the wild-type CDV strain RI.

The organotypic brain slice culture model based on tissues from natural host species offers a new approach to study the virus entry to the brain. However, the protocol does not rule out mechanical trauma and therefore can lead to activation of innate immune and repair responses upon handling. We also observed the disappearance of infected cells over time, but we did not have the means to find out how many non-infected disappeared upon culturing and whether the disappearance of cells is caused by the infection, the culturing condition, or normal cell death. In recent years, improved organotypic brain culture systems have been developed to ensure prolonged survival of the tissues, with significant changes to the classical protocol, which often relies on culturing the slices in medium. This classical protocol results in a metabolic switch from respiration to glycolysis that consequently can play a role in the long-term viability of the tissues. Improvements include the use of low temperature, different culture media, reduced slice thickness, and CSF-like liquid rather than medium [64]. These new methods could improve the study of morbillivirus entry and spread in brain slice cultures.

In conclusion, our study offers a new perspective on the neurotropism and neurovirulence of CDV and MV using organotypic brain cultures obtained from natural host species. We found that microglia and neurons are the potential target cells in morbillivirus virus CNS invasion and despite the differences in neurovirulence in vivo, MV exhibited a surprisingly equivalent level of infection to CDV in the relevant host species. We speculate that the higher level of viraemia during CDV infection as compared to MV infection leads to a higher chance of viral CNS entry and subsequent complications as observed in vivo. Our study also highlights the importance of organotypic brain slice culture in obtaining insights into cellular processes during infection in the CNS and as a useful supplement to in vivo models.

## Figures and Tables

**Table 1:** Semi-quantitative grading of infection levels in ferret and dog tissue slices inoculated with morbilliviruses. Relative morbillivirus infection levels in the olfactory bulb, hippocampal and cortex tissue slices from ferrets (*n* = 3) and dogs (*n* = 3). -: no infected cells; +: low infection level; ++: moderate infection level; +++: high infection level. dpi: days post-inoculation. CDV-RI: rCDV^RI^Venus(6); CDV-SH: rCDV^SH^EGFP(6); MV-KS: rMV^KS^EGFP(3).

Host Species	Virus	Olfactory Bulb			Hippocampus			Cortex		
		1 dpi	2 dpi	3 dpi	1 dpi	2 dpi	3 dpi	1 dpi	2 dpi	3 dpi
Ferret	CDV-RI	-	+	++	-	++	+	-	+	++
	CDV-SH	-	+++	+++	-	+	+	-	++	++
	MV-KS	-	+	+	-	+	+	-	-	+
Dog	CDV-RI	-	++	++	-	-	-	-	+	+
	CDV-SH	-	-	-	-	-	-	-	-	+
	MV-KS	-	++	++	-	-	+	-	+	+

**Table 2:** Semi-quantitative grading of infection levels in NHP brain slice tissues inoculated with morbilliviruses. Relative morbillivirus infection levels in olfactory bulb, hippocampal and cortex tissue slices from rhesus macaques (*n* = 6) -: no cells infected; +: low infection level; ++: moderate infection level; +++: high infection level. dpi: days post-inoculation. MV-KS: rMV^KS^EGFP(3); MV-IC323: rMV^IC323^EGFP(1); CDV-RI: rCDV^RI^Venus(6).

Host Species	Virus	Olfactory Bulb			Hippocampus			Cortex		
		1 dpi	2 dpi	3 dpi	1 dpi	2 dpi	3 dpi	1 dpi	2 dpi	3 dpi
NHP	MV-KS	-	++	++	-	+	++	-	++	+++
	MV-IC323	-	++	++	-	+	++	-	++	+++
	CDV-RI	-	+	-	-	+	-	-	+	-

## Data Availability

The authors declare that all data generated and analysed in this study are included in this article.
